# Efficacy of miRNA-modified mesenchymal stem cell extracellular vesicles in spinal cord injury: A systematic review of the literature and network meta-analysis

**DOI:** 10.3389/fnins.2022.989295

**Published:** 2022-10-05

**Authors:** Zhelun Yang, Jian Rao, Zeyan Liang, Xiongjie Xu, Fabin Lin, Yike Lin, Chunhua Wang, Chunmei Chen

**Affiliations:** Department of Neurosurgery, Affiliated Union Hospital, Fujian Medical University, Fuzhou, China

**Keywords:** spinal cord injury, extracellular vesicles, network meta-analysis, miRNAs, animal

## Abstract

**Background:**

Although some previous studies have indicated that extracellular vesicles (EVs) secreted from miRNA-modified mesenchymal stem cells (MSCs) may be more effective as compared with control EVs in the treatment of rats with spinal cord injuries (SCI), the efficacy of this treatment modality remains controversial.

**Objectives:**

The current study comprehensively evaluated the efficacy of different administered doses of EVs, including miRNA-overexpressing MSCs-derived EVs, among SCI rats. The efficacy of EVs' treatment was evaluated in different SCI models to provide evidence for preclinical trials.

**Methods:**

We extensively searched the following databases to identify relevant studies: PubMed, Embase, Scopus, The Cochrane Library, and Web of Science (from inception to July 20, 2022). Two trained investigators independently screened literature, extracted the data, and evaluated literature quality.

**Results:**

Thirteen studies were included in this network meta-analysis. The results demonstrated that miRNA-overexpressing MSCs-derived EVs (100 and 200 μg of total protein of EVs) significantly improved hind limb motor function in rats at early stages of SCI (i.e., at 3 days after injury) as compared with EVs (100 and 200 μg of total protein of EVs, respectively). However, in the middle and late stages (14 and 28 days), there were no statistically significant differences between EVs with 200 μg dosages and miRNA-loaded EVs with 100 μg dosages. In the late stages (28 days), there were no statistically significant differences between EVs with 100 μg dosages and miRNA-loaded EVs with 200 μg dosages. We found that miRNA-overexpressing MSCs-derived EVs significantly improved motor function among early-stage SCI rats in a compression and contusion model (3 days) as compared with MSCs-derived EVs and miRNA-overexpressing MSCs-derived EVs likewise significantly improved motor function among SCI rats in a contusion model at middle and late stages (14 and 28 days).

**Conclusion:**

Our results suggest that miRNA-overexpressing MSCs-derived EVs (200 μg of total protein of EVs) may be the best choice for the effective treatment of SCI, and miRNA-overexpressing MSCs-derived EVs may likewise be the best choice for treating contusions. However, there are some risks of bias in our included studies, and the mechanisms underlying the efficacy of EVs remain unclear.

**Systematic review registration:** https://www.crd.york.ac.uk/prospero/display_record.php?RecordID=282051, identifier: CRD42021282051.

## Introduction

Spinal cord injury (SCI) is a disease associated with a high disability rate. SCI not only affects patient quality of life, but also imposes a heavy burden on society. Hence, it is essential to develop effective treatments for SCI (Ahuja et al., 2017). Early surgical decompression is considered an effective treatment (Ramakonar and Fehlings, 2021). However, there is currently a lack of effective drugs for inhibiting secondary injury. SCI is characterized by progressive loss of neurologic function, with secondary injuries causing severe damage such as release of toxic chemicals and proinflammatory cytokines, hemorrhage and ischemia of blood vessels, and neuronal apoptosis (McDonald and Sadowsky, 2002). Thus, it is critical to develop effective drugs. Currently, melatonin (Zhang et al., 2018), riluzole (Fehlings et al., 2021a), Rho inhibitors (Fehlings et al., 2021b), and methylprednisolone (Liu et al., 2020) have been evaluated in clinical and preclinical trials. The efficacy of these drugs remains controversial. Generally speaking, the clinical application of new drugs necessitates preclinical trials including drugs for SCI. Currently, an array of new drugs are being developed within animal models of SCI (Kabu et al., 2015).

Recently, various major breakthroughs have been made in cell therapy with respect to treating SCI, including treatment with mesenchymal stem cells (MSCs) (Huang W. et al., 2021). However, immune rejection and tumorigenicity limit the clinical application of cell therapy. Studies suggest that the remarkable effects of cell therapy can be attributed to its dominant paracrine effect (Liu et al., 2021). Extracellular vesicles (EVs), an intercellular communication tool, play a critical role in this putative mechanism. Exosomes, ectosomes or shedding microvesicles (SMVs), and apoptotic bodies are the main subtypes of EVs and differ for their biogenesis, functions and properties. Exosomes are small EVs 40–150 nm in size. Moreover, exosomes are endosome-derived molecules secreted in most cells and are reported to carry proteins, lipids, DNA, and RNAs (Jeppesen et al., 2019). In contrast to exosomes, ectosomes or shedding microvesicles (SMVs) are large vesicles between 100 and 1,000 nm in diameter that are secreted by outward budding of the plasma membrane. Apoptotic bodies are heterogeneous vesicles released from apoptotic cells and are thought to be ~50–5,000 nm in diameter. These vesicles are between 50 and 1,000 nm in size and partially overlap with the exosome size. For this reason, it has not been possible to isolate pure subtypes of EVs to date. For further information on the different origins of these types of vesicles, their content, and their functions, the reader is referred to several comprehensive reviews (Kalra et al., 2016; Thery et al., 2018; van Niel et al., 2022). There are many different sources of EVs, including MSCs, neural stem cells (NSCs) (Rong et al., 2019), macrophage (Zhang B. et al., 2021), pericyte (Yuan et al., 2019), Schwann cells (SCs) (Pan et al., 2021), and olfactory ensheathing cells (OECs) (Tu and Hsueh, 2020). Among them, MSC-derived EVs were widely studied and were considered the most appropriate source (Ren et al., 2020). MSCs originate from bone marrow, adipose tissue, and umbilical cord, having pluripotent differentiation ability and low immunogenicity (Liau et al., 2020). It is reported that MSC-derived EVs could significantly reduce neuronal apoptosis and the expression levels of inflammatory markers TNF-α, IL-1β, and IL-6 (Gu et al., 2020). Moreover, they could inhibit fibrotic and glial scar formation by reducing CSPG deposition and the activation of A1 neurotoxic reactive astrocytes (Liu et al., 2019).

A meta-analysis showed that EVs improve locomotor function in rodents with SCI; however, there are still many controversial issues in the treatment of SCI *via* EVs, especially with respect to differential effects in humans and animal models as well as controversies with regard to the most effective administered EVs' dose (Yi and Wang, 2021). Some studies indicate that EVs secreted from miRNA-modified MSCs are more effective within SCI as compared with control EVs (Huang et al., 2020; Li et al., 2020; Zhang A. et al., 2021). Both miR-181c (Zhang M. et al., 2021) and miR-26a (Chen et al., 2021) can inhibit the expression of the target gene PTEN, which attenuates the inflammatory response. Nevertheless, the efficacy of this treatment modality remains controversial. To the best of our knowledge, there are no trials comparing different EVs' doses and no trials evaluating miRNA-loaded EVs in the treatment of SCI. There are likewise no trials comparing the efficacy of miRNA-loaded and non-miRNA-loaded EVs within different SCI models. MiRNA-modified MSCs are MSCs that were transfected with miRNA mimic in cell culture. EVs derived from MSCs over-expressing a specific miRNA were named miRNA-loaded EVs in the following studies.

Thus, we conducted a systematic review of the literature and network meta-analysis of data from literature research to evaluate the efficacy of different administered EVs' doses and the effects of miRNA-loaded EVs among SCI rats. We likewise evaluated the efficacy of EVs and miRNA-loaded EVs in different SCI models to provide rigorous evidence for preclinical trials.

## Methods

We adhered to PRISMA (Preferred Reporting Items for Systematic Reviews and Meta-Analysis) guidelines in conducting this systematic review (Page et al., 2021). The study protocol was registered in the PROSPERO database with registration number: CRD42021282051. The following search terms were used: (“spinal cord injury” OR “Hemisection” OR “contusion injury” OR “dorsal column injury” OR “complete transection” OR “corticospinal tract injury” OR “Paraplegia” OR “Quadriplegia” OR “Hemiplegia” OR “tetraplegia” OR “Monoplegia” OR “spinal cord trauma” OR “spinal cord transection” OR “spinal cord laceration” OR “spinal cord compromise” OR “spinal cord lesion” OR “spinal cord rupture” OR “spinal cord contusion” OR “spinal cord compression” OR “spinal cord hemisection” OR “traumatic myelopath”) AND (“extracellular vesicles” OR “ exosomes” OR “nano sized vesicles” OR “microvesicles” OR “shedding vesicles” OR “apoptotic bodies”). A total of 197 studies were identified according to search terms. We screened a total of 166 studies, extracted the data of 13 included studies, and evaluated the literature quality of 13 included studies.

### Search strategies

We extensively searched the following databases from inception to July 20, 2022 to identify relevant studies: PubMed, Embase, Scopus, The Cochrane Library, and Web of Science databases. We likewise searched the references within identified reviews and manuscripts. Language restriction was English. The comprehensive search strategy is presented in Supplementary File 1.

### Study selection

Two investigators (ZLY and JR) screened literature separately according to the inclusion and exclusion criteria specified below. If the two researchers had different opinions, a decision was negotiated. If this negotiation was not effective, it was arbitrated by a third party (CMC).

### Eligibility criteria

We developed inclusion and exclusion criteria for this study in strict accordance with PICOS (population, intervention, comparison, outcome, study design) principles. The inclusion and exclusion criteria are as follows.

Types of participants (**P**): All studies on SCI rats were included. Studies in other animals and in humans with SCI were excluded.

Types of interventions (**I**): All studies using EVs derived from miRNA-modified MSCs in the treatment of SCI were included. Studies using EVs derived from cells without miRNA modification were excluded.

Types of comparisons (**C**): Studies in which animals were divided into at least three groups were included. The control group received phosphate buffered saline (PBS) following SCI, and the two intervention groups received EVs and miRNA-loaded EVs, respectively.

Types of outcomes (**O**): The outcome indicator used in this review was the Basso, Beattie & Bresnahan (BBB) scoring system, which can be used to assay the hind limb motor function among rats.

Types of studies (**S**): All studies evaluating miRNA-loaded EVs with respect to improving locomotor function among rats with SCI were included.

### Data extraction and quality assessment

Two trained investigators (ZLY and JR) independently extracted the data from studies screened according to the aforementioned inclusion and exclusion criteria and cross-checked the identified studies. In the case of any disputes, a third party (CMC) was consulted to solve these disputes through negotiation. The extracted contents included (a) study characteristics: author, year, animal models, sex, weight, anesthetics, SCI models, the origins of the EVs, administered doses, and routes of administration; and (b) outcome indicators (i.e., BBB scores). The SYRCLE (Systematic Review Center for Laboratory Animal Experimentation) risk of bias tool was used to assay the quality of the included studies, with respect to selection bias, performance bias, detection bias, attrition bias, reporting bias, and other considerations from a list of 10 entries (Hooijmans et al., 2014).

### Outcome measurements

BBB scores were implemented to assay the hind limb motor function of rats. Rats were placed in an open field and allowed to move freely for 5 min. Two independent researchers blinded to the rats' grouping then recorded the movements of the hip, knee, and ankle joints. The scores ranged from 0 (flaccid paralysis) to 21 (normal gait). The average score of the two observers was used as the final score for each outcome.

### Statistical analysis

An R version 4.1.1 based on Bayesian model (The R Project for Statistical Computing, Vienna, Austria) was used to perform the current network meta-analysis. Mean differences (MD) and associated 95% confidence intervals (CI) were utilized for continuous outcomes. The adjusted indirect comparisons were performed based on MD and 95% CI values to assess the indirect comparisons with respect to the efficacy of different animal models and the administered EVs' dose. *I*^2^ tests were used to assess statistical heterogeneity. *I*^2^ values >50% were considered statistically significant heterogeneity. A random-effects model was used in the current study. The results are shown *via* forest plots. The surface under the cumulative ranking curve (SUCRA) was used to estimate ranking probabilities (Salanti et al., 2011).

There are three key assumptions within network meta-analysis: similarity, transitivity, and consistency (Cipriani et al., 2013). The inclusion and exclusion criteria were tightly controlled to meet the similarity and transitivity assumptions, including with respect to the severity of illness at baseline, sample size, sex, weight, EVs' origins, and routes of administration (Salanti, 2012). The animal models and interventions in all included studies within the network meta-analysis were likewise evaluated *via* meta-regression. By definition, inconsistency could not occur within a multi-arm study (Higgins et al., 2012). Goodness-of-fit was assessed *via* the deviance information criterion (DIC). Model fit was evaluated by comparing the DIC values between the consistency and inconsistency models.

## Results

### Article selection process

**Figure 1:**
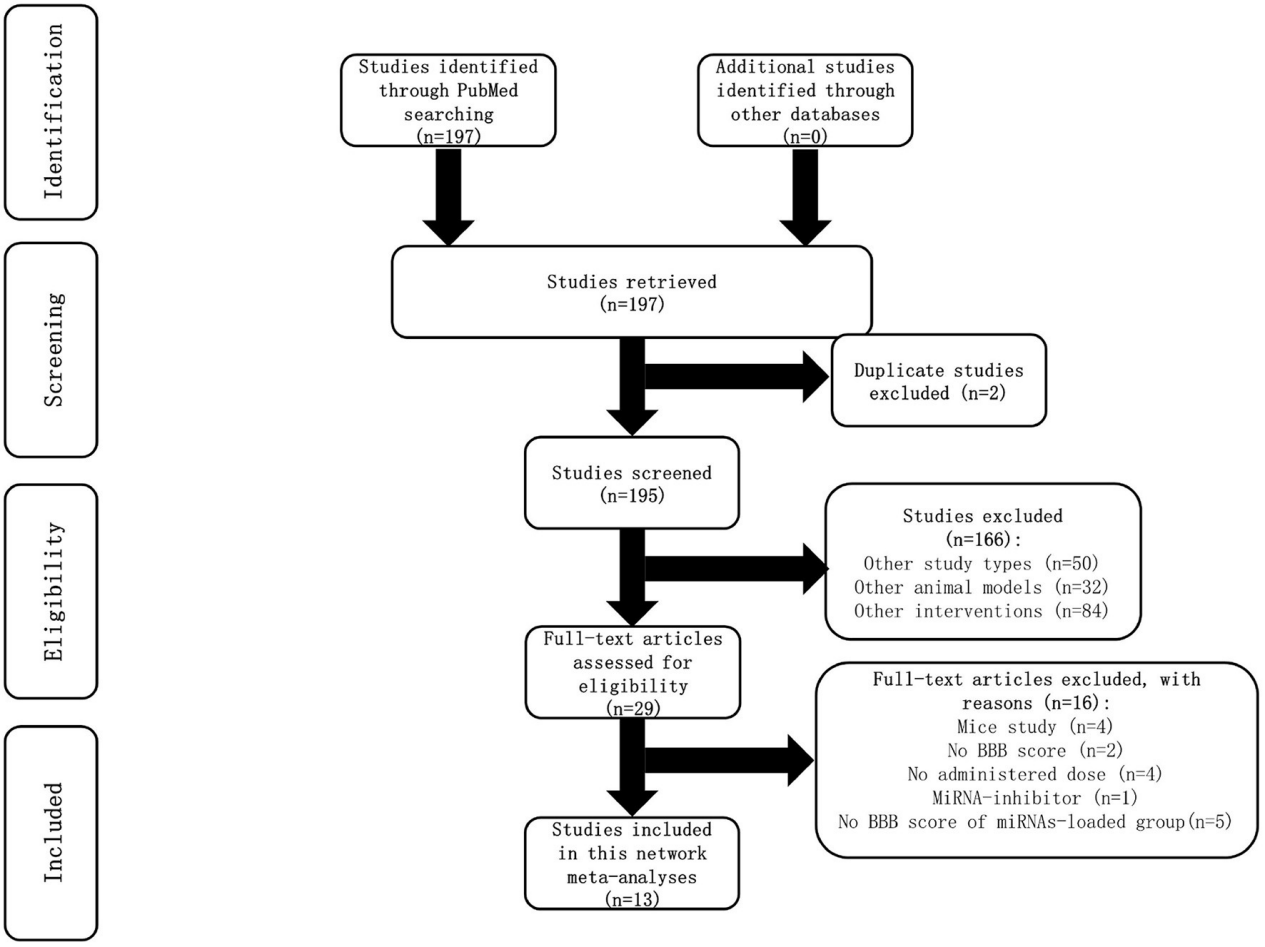
Flow chart of article selection.

A total of 197 studies were identified in searching the PubMed database. However, no relevant studies were found from other databases (Supplementary File 1). Two duplicate studies were excluded. After screening the title and abstract, 166 studies were removed because of the study types, animal models, and interventions. The full text of the remaining 29 studies was meticulously retrieved for assessment; 16 studies were excluded due to reasons specified in the flow chart (Figure 1). A total of 13 studies were included in this network meta-analysis.

### Study characteristics

The characteristics of the 13 included studies are summarized in Table 1. The sample size for each group ranged from *n* = 6 to *n* = 24. Most (92.3%) animal models were Sprague Dawley (SD) rats. Most (69.23%) were male. The body weights ranged from 80 to 300 g, and ages varied from 2 to 8 weeks. The SCI models included transection, contusion, and compression models. All studies assessed the thoracic spinal cord (T10) level at which damaged. Compression injury was induced using an aneurysm clip in two studies, and contusion injury was impacted using a standard striking device in four studies. In all these studies, all EVs originated from mesenchymal stem cells (MSCs) and almost all EVs were injected through the tail vein. Three studies used the ExoQuick-TC kit, and 10 used ultracentrifugations to isolate EVs. Ultimately, the administered EVs' dose ranged from 100 to 700 μg. In nine studies, the time of dosing was delayed (i.e., administration within 24 h after surgery). In four studies, the EV was administered immediately after surgery. Although EVs derived from MSCs overexpress specific miRNAs (miR-181c, miR-26a, miR-126, miR-29b, miR-133b, miR-544, miR-494, miR-329-3p, miR-29b, miR-511-3p, miR-22, miR-9-5p, and let-7a-5p) differently, their role in promoting SCI repair is the same. The MSCs were transfected with miRNA with Lipofectamine in eight studies, with siRNA in three studies, and with Lentiviral vector in one study. However, there is one study that did not specify the method.

**Table 1:** Characteristics of included studies.

**References**	**Country**	**Journal**	**The sample size of each group**	**Animal**	**Sex**	**Age**	**Weight**	**Anesthetic**	**SCI model**	**miRNA**	**The method of miRNA overexpression in MSCs**	**The origin of EVs**	**EV extraction method**	**The period of treatment**	**Administered dose**	**The route of administration**	**Outcomes**
Zhang M. et al. (2021)	China	Journal of molecular histology	8	SD rats	Male	8 weeks	200–230 g	10% chloral hydrate (3 mg/kg)	T10 contusion	miR-181c	siRNA	BMSCs	Ultracentrifugation	30 min, 7 days, and 14 days after surgery	200 μg each time	Tail vein injection	BBB
Chen et al. (2021)	China	Stem cell research & therapy	6	SD rats	Male	6–8 weeks	Not described	2.5–3% isoflurane	T10 compression	miR-26a	Lipofectamine 3000	BMSCs	Ultracentrifugation	Immediately after surgery	200 μg	Tail vein injection	BBB
Huang et al. (2020)	China	Neuroscience	10	SD rats	Male	Not described	180–220 g	10% chloral hydrate (3 mg/kg)	T10 contusion	miR-126	Lipofectamine 3000	BMSCs	Ultracentrifugation	30 min after surgery	100 μg	Tail vein injection	BBB
Yu et al. (2019)	China	Brazilian journal of medical and biological research	20	SD rats	Female	Not described	230–250 g	1% pentobarbital sodium (50 mg/kg)	T10 contusion	miR-29b	Lentiviral vector (LV)	BMSCs	ExoQuick-TC kit	1 h after surgery	100 μg	Tail vein injection	BBB
Li et al. (2018)	China	Frontiers in neuroscience	6	SD rats	Male	Not described	250–300 g	Chloral hydrate (400 mg/kg)	T10 compression	miR-133b	Lipofectamine 3000	BMSCs	ExoQuick-TC kit	24 h after surgery	100 μg	Tail vein injection	BBB
Li et al. (2020)	China	Archives of physiology and biochemistry	10	SD rats	Male	8 weeks	Not described	10% chloral hydrate (3 mL/kg)	T10 contusion	miR-544	Lipofectamine 3000	BMSCs	Ultracentrifugation	24 h after surgery	100 μg	Tail vein injection	BBB
Huang W. et al. (2021)	China	Oxidative Medicine and Cellular Longevity	6	SD rats	Male	2 weeks	80–100 g	4% sodium pen tobarbital (50 mg/kg)	T10 contusion	miR-494	siRNA	BMSCs	ExoQuick-TC kit	Per 24 h for 7 consecutive days after surgery	100 μg each time	Tail vein injection	BBB
Liu et al. (2022)	China	Journal of Molecular Neuroscience	6	SD rats	Male	6–8 weeks	250–300 g	10% chloral hydrate (400 mg/kg)	T10 contusion	miR-329-3p	Lipofectamine 3000	BMSCs	Ultracentrifugation	Immediately after surgery	100 μg	Tail vein injection	BBB
Nie and Jiang (2021)	China	Bioengineered	24	SD rats	Male	Not described	180–220 g	2% sodium pen tobarbital (40 mg/kg)	T10 transection	miR-23b	Lipofectamine 3000	BMSCs	Ultracentrifugation	24 h after surgery	100 μg	Tail vein injection	BBB
Huang et al. (2022)	China	Brain Research Bulletin	10	SD rats	Not described	8 weeks	200–220 g	Sodium pen tobarbital (65 mg/kg)	T10 contusion	miR-511-3p	Lipofectamine 2000	AD-MSCs	Ultracentrifugation	Immediately after surgery	200 μg	Tail vein injection	BBB
Sheng et al. (2021)	China	Journal of cellular and molecular medicine	6	SD rats	Male	6 weeks	Not described	Chloral hydrate (4 mg/kg)	T10 compression	miR-22	Not described	BMSCs	Ultracentrifugation	One day before surgery, every 3 days for 15 continuous days after surgery	100 μg each time	Tail vein injection	BBB
He et al. (2022)	China	Molecular immunology	10	SD rats	Not described	6 weeks	220 g	1% sodium pentobarbital (50 mg/kg)	T10 contusion	miR-9-5p	Lipofectamine 3000	BMSCs	Ultracentrifugation	Every day for consecutive 7 days after surgery	100 μg each time	Tail vein injection	BBB
Wang et al. (2022)	China	Frontiers in molecular neuroscience	10	Wistar rats	Female	6–8 weeks	200–250 g	Ketamine (60 mg/kg) and xylazine (5 mg/kg)	T10 contusion	let-7a-5p	siRNA	BMSCs	Ultracentrifugation	Immediately continuous injection for 3 days after surgery	25 μl	Intrathecal injection	BBB

### Quality assessment and publication bias

**Figure 2:**
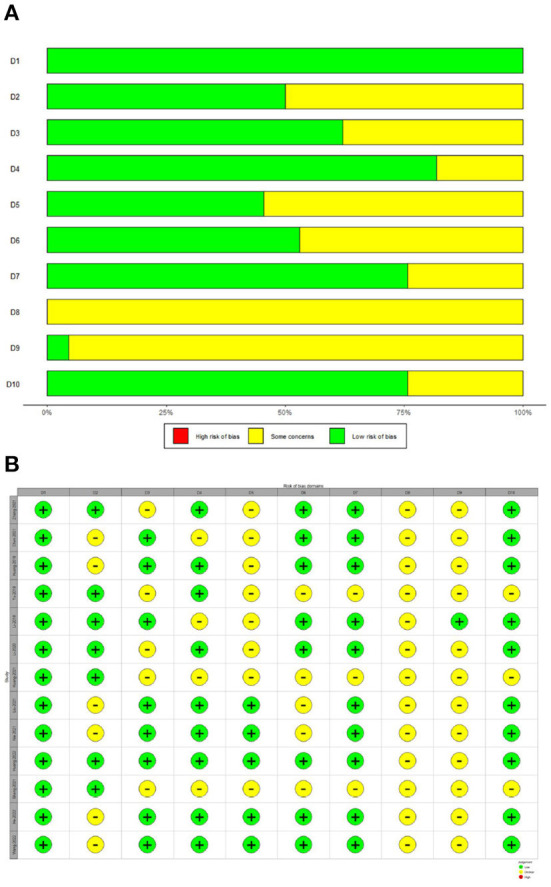
Risk of bias with SYRCLE tool. **(A)** Risk of bias graph. **(B)** Risk of bias summary. D1 (Selection bias): Was the allocation sequence adequately generated and applied? D2 (Selection bias): Were the groups similar at baseline or were they adjusted for confounders in the analysis? D3 (Selection bias): Was the allocation adequately concealed? D4 (Performance bias): Were the animals randomly housed during the experiment? D5 (Performance bias): Were the caregivers and/or investigators blinded from knowledge which intervention each animal received during the experiment? D6 (Detection bias): Were animals selected at random for outcome assessment? D7 (Detection bias): Was the outcome assessor blinded? D8 (Attrition bias): Were incomplete outcome data adequately addressed? D9 (Reporting bias): Are reports of the study free of selective outcome reporting? D10 (Other): Was the study apparently free of other problems that could result in high risk of bias?

**Figure 3:**
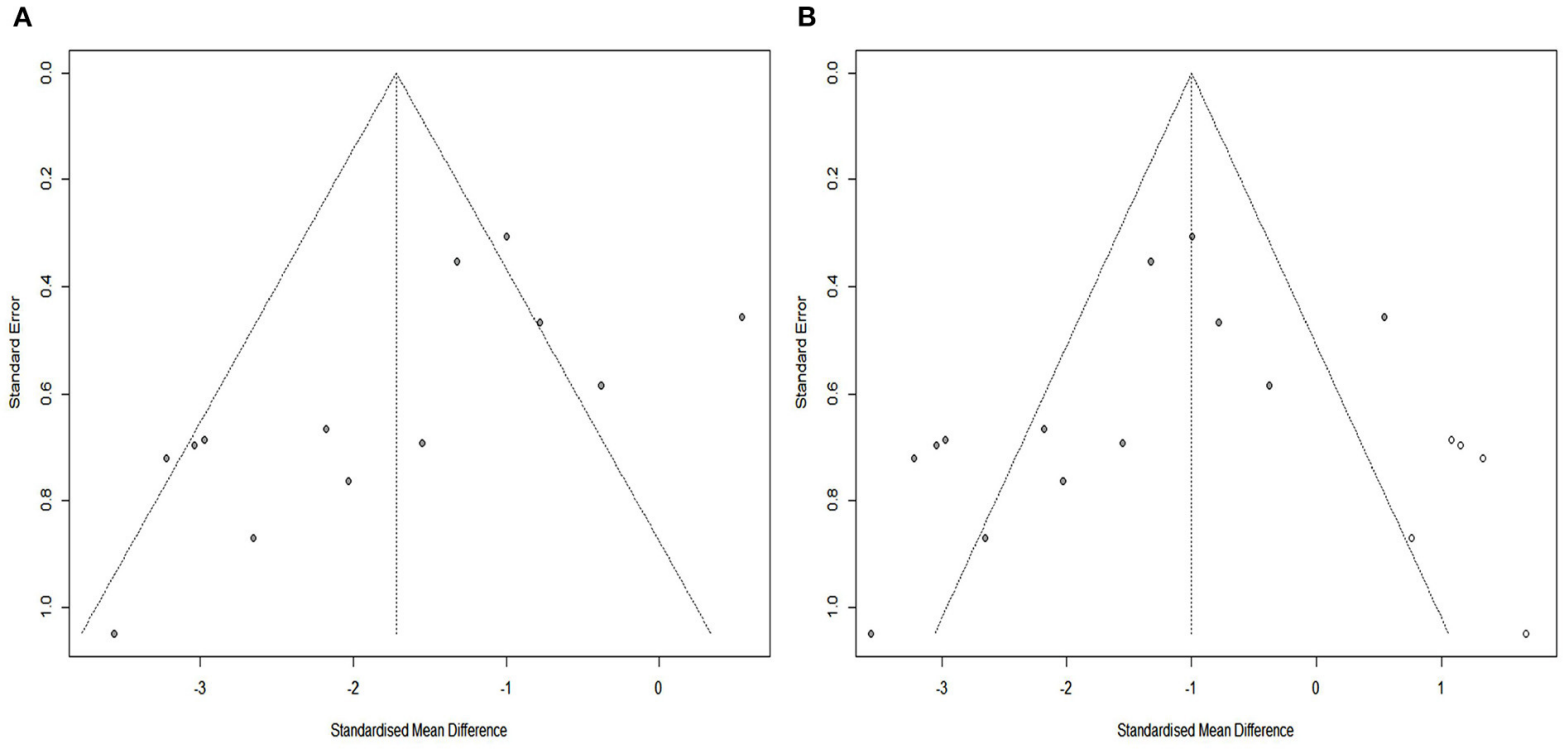
Assessment of publication bias. **(A)** Funnel plots showed pronounced asymmetry. **(B)** Trim-and-fill analysis predicted 5 “missing” studies (unfilled circles).

The SYRCLE tool was used to assay the risk of bias in all included studies. The methodologic quality of the results from each study are summarized in Figure 2. There was no high risk of bias in the 13 included studies. All included studies had a low risk of sequence generation with respect to selection bias. However, all included studies had an unclear risk of incomplete outcome data for attrition bias and 12 (92%) had an unclear risk of selective outcomes for reporting bias. Moreover, most included studies showed low risk with respect to many items, including allocation concealment in terms of selection bias, baseline characteristics for selection bias, random animal housing and blinding with regard to performance bias, random outcome assessment and blinding for detection bias, and other sources of bias. The methodologic quality of all the included trials was satisfactory. According to Cochrane Group guidelines, funnel plots are not recommended for detecting publication bias when fewer than 10 studies are included in a meta-analysis (Stang, 2010). However, this study included 13 studies. Therefore, we used funnel plots and Egger regression tests to assess publication bias. As shown in the Figure 3A, there is a significant funnel plot asymmetry visually, indicating publication bias. Egger's test (*P* = 0.0217) was used to test for potential publication bias in the primary results. The trim and fill analysis (Figure 3B) estimated five “missing” unpublished studies on the right side of the funnel plot.

### Network meta-analysis of BBB scores at different dosages

**Figure 4:**
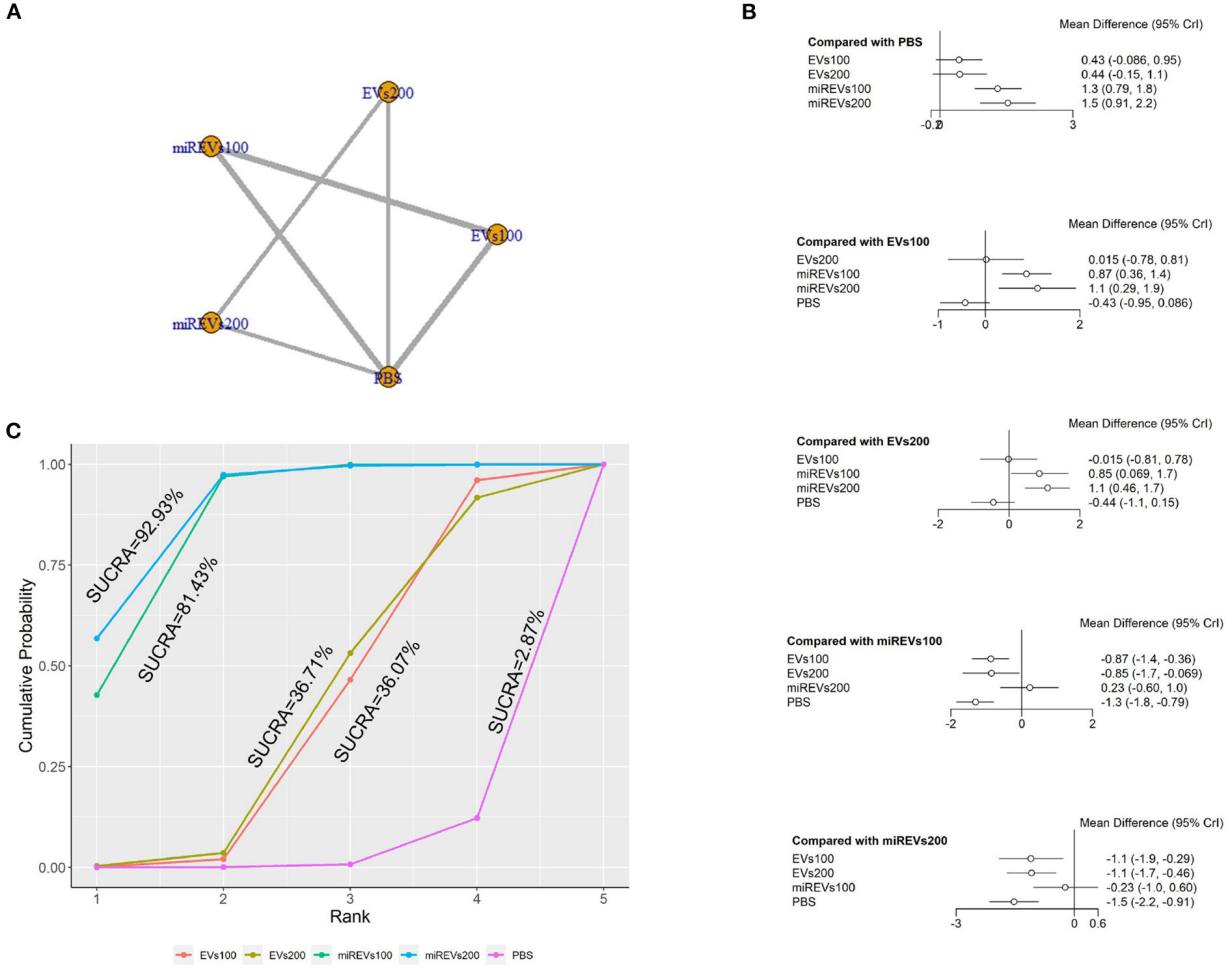
The network meta-analysis of BBB scores at different dosages at 3 days after administration. **(A)** Network plot; **(B)** Forest plot; **(C)** SUCRA plot. EVs100, EVs at 100 μg dosages; EVs200, EVs at 200 μg dosages; miREVs100, miRNAs-loaded EVs at 100 μg dosages; miREVs200, miRNAs-loaded EVs at 200 μg dosages.

First, with respect to findings 3 days after administration, one study (Wang et al., 2022) was excluded because a polyethylene catheter was placed at the injured level for continuous intrathecal injection and two studies (Huang W. et al., 2021; He et al., 2022) were excluded because the administered dose had changed at 3 days. Therefore, 10 included studies (Li et al., 2018, 2020; Yu et al., 2019; Huang et al., 2020, 2022; Chen et al., 2021; Nie and Jiang, 2021; Sheng et al., 2021; Zhang M. et al., 2021; Liu et al., 2022) were used for the data analysis. The results of this network meta-analysis suggest that the number of studies evaluating EVs and miRNA-loaded EVs was higher with the 100 μg dose as compared with the 200 μg dose (Figure 4A). Moreover, forest plots (Figure 4B) and league tables (Table 2) show that miRNA-loaded EVs at 100 μg dosages presented with a statistically significant advantage over PBS (MD = 1.3; 95% CI: 0.79, 1.84; *p* < 0.01) and EVs at 100 μg dosages (MD = 0.87; 95% CI: 0.36, 1.39; *p* < 0.01) and 200 μg dosages (MD = 0.86; 95% CI: 0.07, 1.67; *p* < 0.01), respectively. Additionally, miRNA-loaded EVs at 200 μg dosages presented with a statistically significant advantage over PBS (MD = 1.53; 95% CI: 0.91, 2.15; *p* < 0.01) and EVs at 100 μg dosages (MD = 1.1; 95% CI: 0.29, 1.9; *p* < 0.01) and 200 μg dosages (MD = 1.09; 95% CI: 0.47, 1.71; *p* < 0.01), respectively. However, the differences were not statistically significant with respect to the other comparisons. Finally, the SUCRA ranking graph showed that miRNA-loaded EVs at 200 μg dosages (92.93%) ranked highest, followed by miRNA-loaded EVs at 100 μg dosages (81.43%), EVs at 200 μg dosages (36.71%), EVs at 100 μg dosages (36.07%), and PBS (2.87%) (Figure 4C).

**Table 2:** League table of BBB scores at different dosages at 3 days after administration.

EVs100	0.01 (−0.79, 0.8)	0.87 (0.36, 1.39)	1.1 (0.29, 1.9)	−0.43 (−0.95, 0.08)
−0.01 (−0.8, 0.79)	EVs200	0.86 (0.07, 1.67)	1.09 (0.47, 1.71)	−0.44 (−1.05, 0.16)
−0.87 (−1.39, −0.36)	−0.86 (−1.67, −0.07)	miREVs100	0.23 (−0.59, 1.03)	−1.3 (−1.84, −0.79)
−1.1 (−1.9, −0.29)	−1.09 (−1.71, −0.47)	−0.23 (−1.03, 0.59)	miREVs200	−1.53 (−2.15, −0.91)
0.43 (−0.08, 0.95)	0.44 (−0.16, 1.05)	1.3 (0.79, 1.84)	1.53 (0.91, 2.15)	PBS

The gray color shades represent the different interventions and the bold values represent statistical significance.

**Figure 5:**
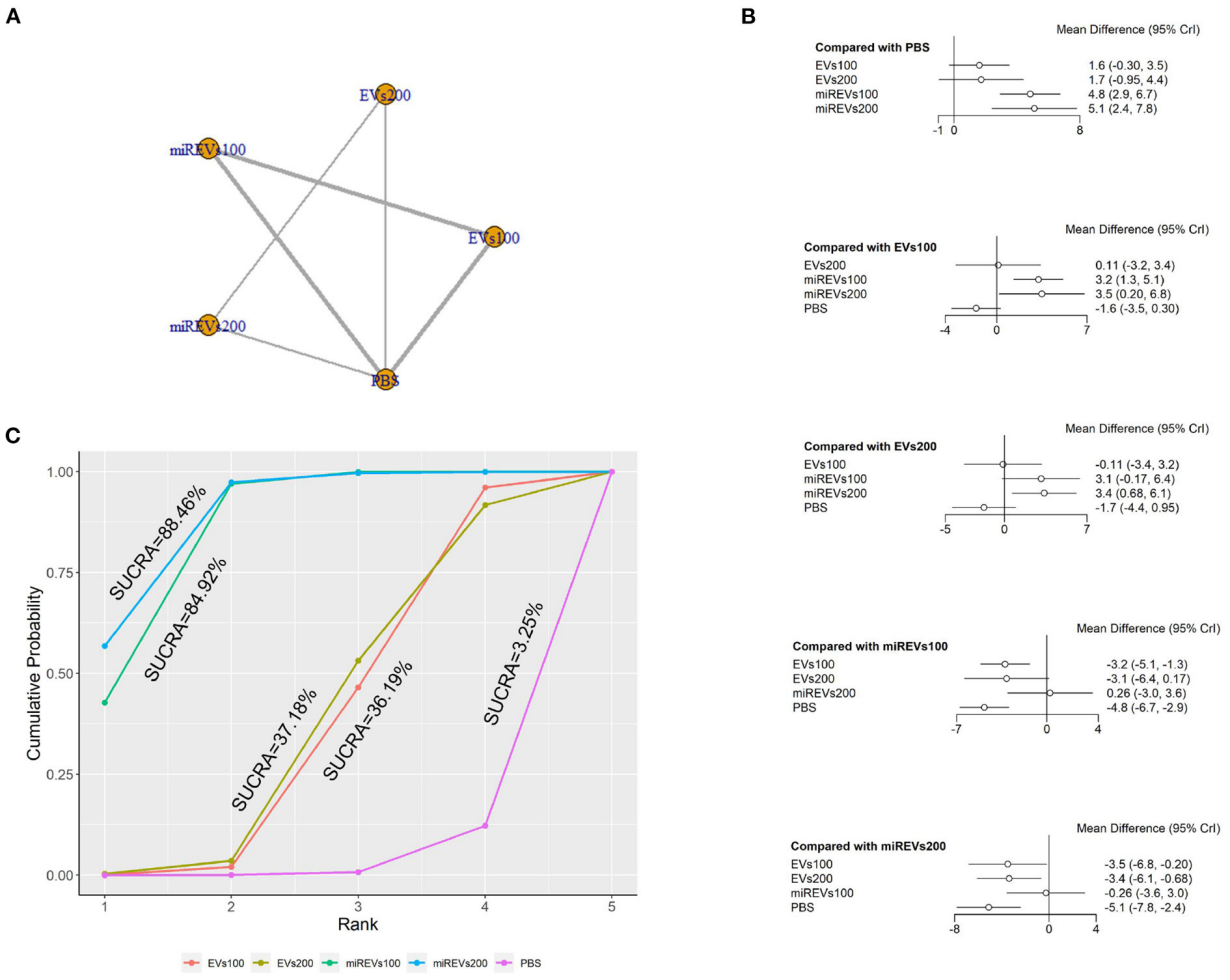
The network meta-analysis of BBB scores at different dosages at 14 days after administration. **(A)** Network plot; **(B)** Forest plot; **(C)** SUCRA plot. EVs100, EVs at 100 μg dosages; EVs200, EVs at 200 μg dosages; miRExos100, miRNAs-loaded EVs at 100 μg dosages; miREVs200, miRNAs-loaded EVs at 200 μg dosages.

Second, with respect to findings 14 days after administration, two studies (Li et al., 2020; Liu et al., 2022) were excluded because BBB scores were not assessed at 14 days and four studies (Huang W. et al., 2021; Sheng et al., 2021; Zhang M. et al., 2021) were excluded because the administered dose had changed at 14 days. Furthermore, one study (Wang et al., 2022) was excluded because a polyethylene catheter was placed at the injured level for continuous intrathecal injection. Therefore, six studies (Li et al., 2018; Yu et al., 2019; Huang et al., 2020, 2022; Chen et al., 2021; Nie and Jiang, 2021) were included for data analysis. The results of this network meta-analysis suggest that the number of studies with EVs and miRNA-loaded EVs was higher with the 100 μg dose as compared with the 200 μg dose (Figure 5A). The forest plot (Figure 5B) and league table (Table 3) show that miRNA-loaded EVs at 100 μg dosages presented with a statistically significant advantage over PBS (MD = 4.85; 95% CI: 2.94, 6.74; *p* < 0.01) and EVs at 100 μg dosages (MD = 3.24; 95% CI: 1.33, 5.13; *p* < 0.01), respectively. Additionally, miRNA-loaded EVs at 200 μg dosages presented with a statistically significant advantage over PBS (MD = 5.11; 95% CI: 2.42, 7.79; *p* < 0.01) and EVs at 100 μg dosages (MD = 3.5; 95% CI: 0.2, 6.78; *p* < 0.01) and 200 μg dosages (MD = 3.38; 95% CI: 0.68, 6.09; *p* < 0.01), respectively. However, the differences were not statistically significant among the other comparisons. The SUCRA ranking graph showed that miRNA-loaded EVs at 200 μg dosages (88.46%) ranked highest, followed by the miRNA-loaded EVs at 100 μg dosages (84.92%), EVs at 200 μg dosages (37.18%), EVs at 100 μg dosages (36.19%), and PBS (3.25%) (Figure 5C).

**Table 3:** League table of BBB scores at different dosages at 14 days after administration.

EVs100	0.11 (−3.17, 3.4)	3.24 (1.33, 5.13)	3.5 (0.2, 6.78)	−1.61 (−3.51, 0.3)
−0.11 (−3.4, 3.17)	EVs200	3.12 (−0.17, 6.39)	3.38 (0.68, 6.09)	−1.73 (−4.41, 0.95)
−3.24 (−5.13, −1.33)	−3.12 (−6.39, 0.17)	miREVs100	0.26 (−3.03, 3.56)	−4.85 (−6.74, −2.94)
−3.5 (−6.78, −0.2)	−3.38 (−6.09, −0.68)	−0.26 (−3.56, 3.03)	miREVs200	−5.11 (−7.79, −2.42)
1.61 (−0.3, 3.51)	1.73 (−0.95, 4.41)	4.85 (2.94, 6.74)	5.11 (2.42, 7.79)	PBS

The gray color shades represent the different interventions and the bold values represent statistical significance.

**Figure 6:**
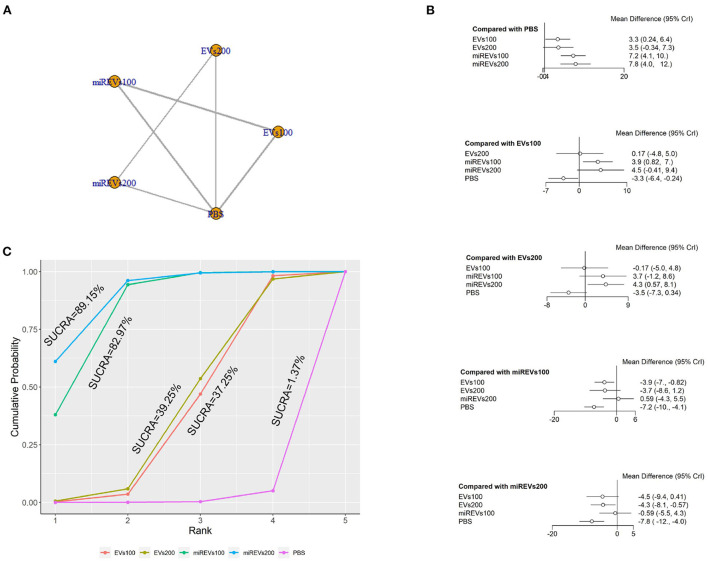
The network meta-analysis of BBB scores at different dosages at 28 days after administration. **(A)** Network plot; **(B)** Forest plot; **(C)** SUCRA plot. EVs100, EVs at 100 μg dosages; EVs200, EVs at 200 μg dosages; miREVs100, miRNAs-loaded EVs at 100 μg dosages; miREVs200, miRNAs-loaded EVs at 200 μg dosages.

Finally, with respect to findings 28 days after administration, three studies (Li et al., 2018, 2020; Liu et al., 2022) were excluded because BBB scores were not assessed at 28 days after administration and four studies (Huang W. et al., 2021; Sheng et al., 2021; Zhang A. et al., 2021; He et al., 2022) were excluded because the administered dose had changed at 28 days. Moreover, one study (Wang et al., 2022) was excluded because a polyethylene catheter was placed at the injured level for continuous intrathecal injection. Thus, five studies (Yu et al., 2019; Huang et al., 2020, 2022; Chen et al., 2021; Nie and Jiang, 2021) were included for data analysis. The results of this network meta-analysis suggest that the number of studies evaluating EVs and miRNAs-loaded EVs was higher with the 100 μg dose than with the 200 μg dose (Figure 6A). The forest plot (Figure 6B) and league table (Table 4) show that miRNA-loaded EVs at 100 μg dosages presented with a statistically significant advantage over PBS (MD = 7.22; 95% CI: 4.14, 10.29; *p* < 0.01) and EVs at 100 μg dosages (MD = 3.9; 95% CI: 0.82, 6.98; *p* < 0.01), respectively. Additionally, miRNA-loaded EVs at 200 μg dosages presented with a statistically significant advantage over PBS (MD = 7.81; 95% CI: 4.03, 11.57; *p* < 0.01) and EVs at 200 μg dosages (MD = 4.33; 95% CI: 0.57, 8.12; *p* < 0.01), respectively. Moreover, EVs at 100 μg dosages presented with a statistically significant advantage over PBS (MD = 3.31; 95% CI: 0.24, 6.42; *p* < 0.01). However, the differences were not statistically significant among the other comparisons. The SUCRA ranking graph showed that miRNA-loaded EVs at 200 μg dosages (89.15%) ranked highest, followed by the miRNA-loaded EVs at 100 μg dosages (82.97%), EVs at 200 μg dosages (39.25%), EVs at 100 μg dosages (37.25%), and PBS (1.37%) (Figure 6C).

**Table 4:** League table of BBB scores at different dosages at 28 days after administration.

EVs100	0.17 (−4.75, 5.04)	3.9 (0.82, 6.98)	4.5 (−0.41, 9.36)	−3.31 (−6.42, −0.24)
−0.17 (−5.04, 4.75)	EVs200	3.74 (−1.15, 8.63)	4.33 (0.57, 8.12)	−3.48 (−7.26, 0.34)
−3.9 (−6.98, −0.82)	−3.74 (−8.63, 1.15)	miREVs100	0.59 (−4.32, 5.46)	−7.22 (−10.29, −4.14)
−4.5 (−9.36, 0.41)	−4.33 (−8.12, −0.57)	−0.59 (−5.46, 4.32)	miREVs200	−7.81 (−11.57, −4.03)
3.31 (0.24, 6.42)	3.48 (−0.34, 7.26)	7.22 (4.14, 10.29)	7.81 (4.03, 11.57)	PBS

The gray color shades represent the different interventions and the bold values represent statistical significance.

### The network meta-analysis of BBB scores in different models

**Figure 7:**
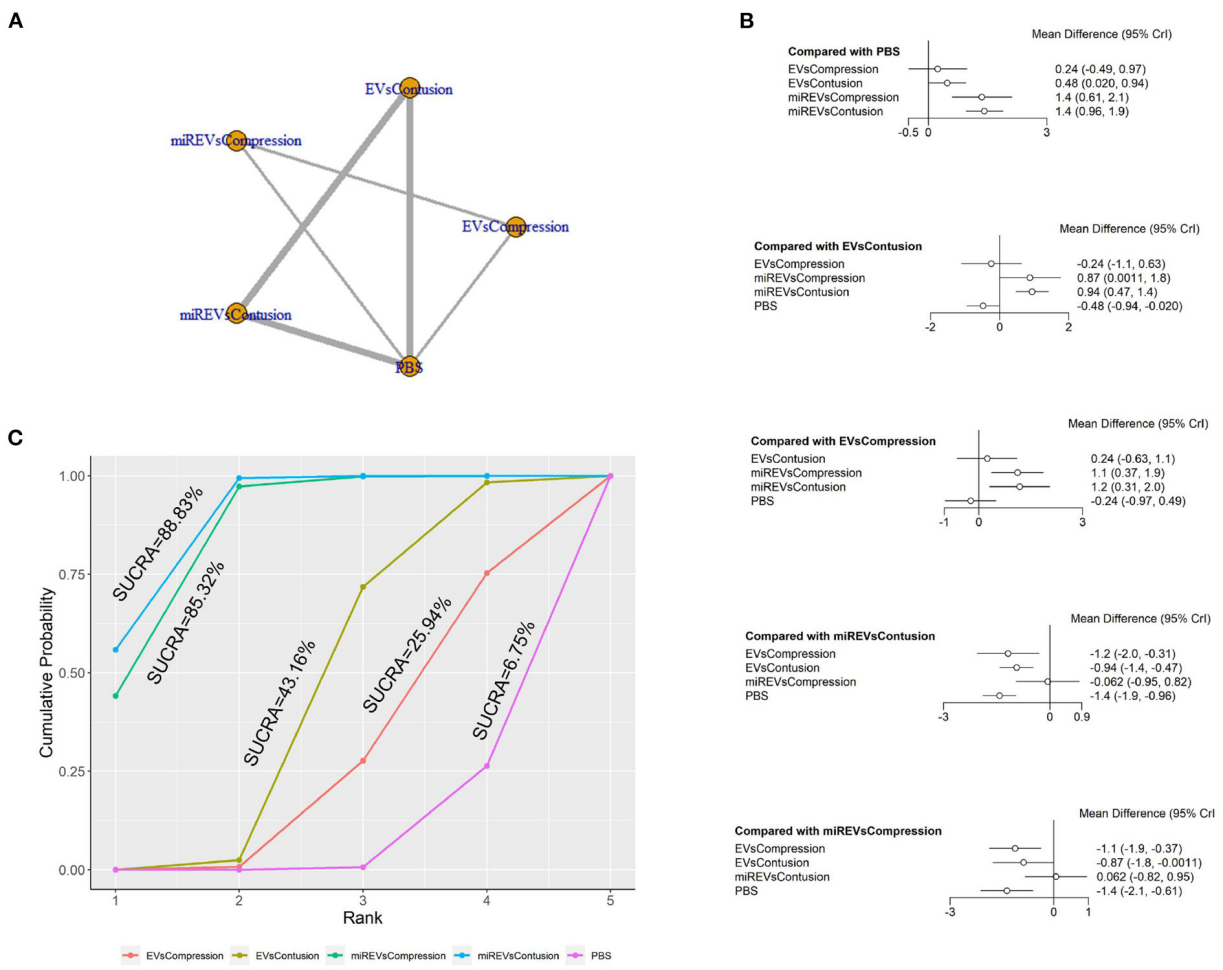
The network meta-analysis of BBB scores in different models at 3 days after administration. **(A)** Network plot; **(B)** Forest plot; **(C)** SUCRA plot. EVsCompression, EVs in compression models; EVsContusion, EVs in contusion models; miREVsCompression, miRNAs-loaded EVs in compression models; miREVsContusion, miRNAs-loaded EVs in contusion models.

First, with respect to findings 3 days after administration, two studies (Huang W. et al., 2021; Wang et al., 2022) were excluded because BBB scores were not assessed at 3 days and one study (Nie and Jiang, 2021) was excluded because the SCI model was transection model. Therefore, 10 included studies (Li et al., 2018, 2020; Yu et al., 2019; Huang et al., 2020, 2022; Chen et al., 2021; Sheng et al., 2021; Zhang M. et al., 2021; He et al., 2022; Liu et al., 2022) were included for data analysis. The results of this network meta-analysis suggest that EVs and miRNA-loaded EVs in contusion models had more studies than in compression models (Figure 7A). Moreover, the forest plot (Figure 7B) and league table (Table 5) showed that the miRNA-loaded EVs in contusion models presented with a statistically significant advantage over PBS (MD = 1.42; 95% CI: 0.96, 1.89; *p* < 0.01) and the EVs in compression models (MD = 1.18; 95% CI: 0.31, 2.05; *p* < 0.01) and contusion models (MD = 0.94; 95% CI: 0.47, 1.41; *p* < 0.01), respectively. Additionally, the miRNAs-loaded EVs in compression models likewise had a statistically significant advantage over PBS (MD = 1.35; 95% CI: 0.61, 2.11; *p* < 0.01) and EVs in compression models (MD = 1.12; 95% CI: 0.37, 1.86; *p* < 0.01) and contusion models (MD = 0.87; 95% CI: 0, 1.76; *p* < 0.01), respectively. Furthermore, EVs in contusion models presented with a statistically significant advantage over PBS (MD = 0.48; 95% CI: 0.02, 0.94; *p* < 0.01). However, the differences were not statistically significant among the other comparisons. Finally, the SUCRA ranking graph showed that miRNA-loaded EVs in contusion models (88.83%) ranked highest, followed by the miRNA-loaded EVs in compression models (85.32%), EVs in contusion models (43.16%), EVs in compression models (25.94%), and PBS (6.75%) (Figure 7C).

**Table 5:** League table of BBB scores in different models at 3 days after administration.

EVsCompression	0.24 (−0.63, 1.1)	1.12 (0.37, 1.86)	1.18 (0.31, 2.05)	−0.24 (−0.97, 0.49)
−0.24 (−1.1, 0.63)	EVsContusion	0.87 (0, 1.76)	0.94 (0.47, 1.41)	−0.48 (−0.94, −0.02)
−1.12 (−1.86, −0.37)	−0.87 (−1.76, 0)	miREVsCompression	0.06 (−0.82, 0.95)	−1.35 (−2.11, −0.61)
−1.18 (−2.05, −0.31)	−0.94 (−1.41, −0.47)	−0.06 (−0.95, 0.82)	miREVsContusion	−1.42 (−1.89, −0.96)
0.24 (−0.49, 0.97)	0.48 (0.02, 0.94)	1.35 (0.61, 2.11)	1.42 (0.96, 1.89)	PBS

The gray color shades represent the different interventions and the bold values represent statistical significance.

**Figure 8:**
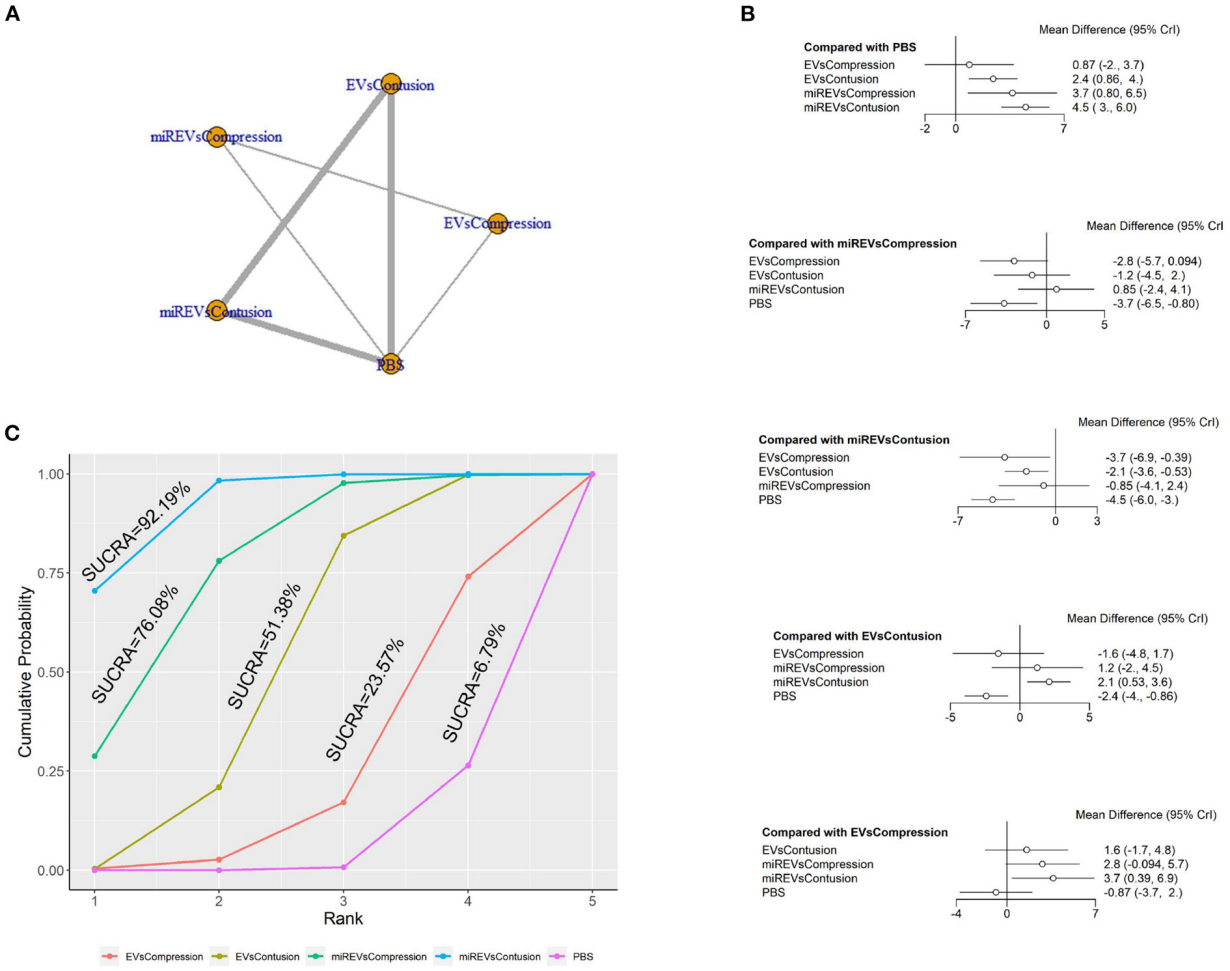
The network meta-analysis of BBB scores in different models at 14 days after administration. **(A)** Network plot; **(B)** Forest plot; **(C)** SUCRA plot. EVsCompression, EVs in compression models; EVsContusion, EVs in contusion models; miREVsCompression, miRNAs-loaded EVs in compression models; miREVsContusion, miRNAs-loaded EVs in contusion models.

Next, with respect to findings 14 days after administration, three studies (Li et al., 2020; Sheng et al., 2021; Liu et al., 2022) were excluded because BBB scores were not assessed at 14 days and one study (Nie and Jiang, 2021) was excluded because the SCI model was transection model. Therefore, a total of nine studies (Li et al., 2018; Yu et al., 2019; Huang et al., 2020, 2022; Chen et al., 2021; Huang L. et al., 2021; Zhang M. et al., 2021; He et al., 2022; Wang et al., 2022) were included for data analysis. The results of this network meta-analysis suggest that EVs and miRNA-loaded EVs had more studies within contusion models as compared with compression models (Figure 8A). The forest plot (Figure 8B) and league table (Table 6) showed that EVs in contusion models (MD = 2.42; 95% CI: 0.86, 3.96; *p* < 0.01) and miRNA-loaded EVs in compression models (MD = 3.66; 95% CI: 0.8, 6.53; *p* < 0.01) and contusion models (MD = 4.52; 95% CI: 2.95, 6.04; *p* < 0.01) presented with a statistically significant advantage over PBS and that miRNA-loaded EVs in contusion models had a statistically significant advantage over the EVs in compression models (MD = 3.65; 95% CI: 0.39, 6.89; *p* < 0.01) and contusion models (MD = 2.1; 95% CI: 0.53, 3.62; *p* < 0.01). The differences were not statistically significant among the other comparisons. The SUCRA ranking graph showed that the miRNA-loaded EVs in contusion models (92.19%) ranked highest, followed by the miRNA-loaded EVs in compression models (76.08%), EVs in contusion models (51.38%), EVs in compression models (23.57%), and PBS (6.79%) (Figure 8C).

**Table 6:** League table of BBB scores in different models at 14 days after administration.

EVsCompression	1.55 (−1.71, 4.81)	2.8 (−0.09, 5.72)	3.65 (0.39, 6.89)	−0.87 (−3.72, 1.99)
−1.55 (−4.81, 1.71)	EVsContusion	1.24 (−2, 4.51)	2.1 (0.53, 3.62)	−2.42 (−3.96, −0.86)
−2.8 (−5.72, 0.09)	−1.24 (−4.51, 2)	miREVsCompression	0.85 (−2.42, 4.07)	−3.66 (−6.53, −0.8)
−3.65 (−6.89, −0.39)	−2.1 (−3.62, −0.53)	−0.85 (−4.07, 2.42)	miREVsContusion	−4.52 (−6.04, −2.95)
0.87 (−1.99, 3.72)	2.42 (0.86, 3.96)	3.66 (0.8, 6.53)	4.52 (2.95, 6.04)	PBS

The gray color shades represent the different interventions and the bold values represent statistical significance.

**Figure 9:**
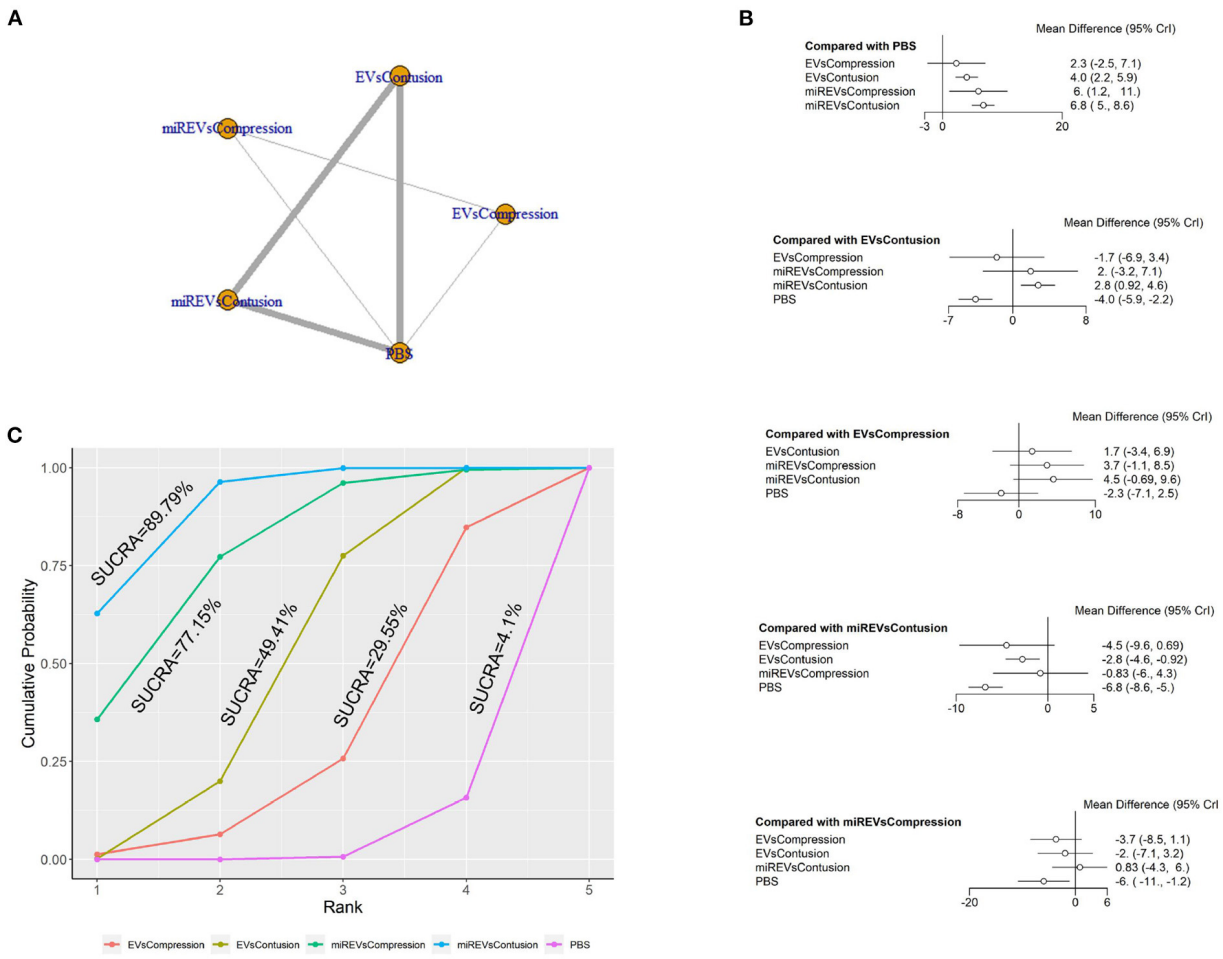
The network meta-analysis of BBB scores in different models at 28 days after administration. **(A)** Network plot; **(B)** Forest plot; **(C)** SUCRA plot. EVsCompression, EVs in compression models; EVsContusion, EVs in contusion models; miREVsCompression, miRNAs-loaded EVs in compression models; miREVsContusion, miRNAs-loaded EVs in contusion models.

Finally, with respect to findings 28 days after administration, four studies (Li et al., 2018, 2020; Sheng et al., 2021; Liu et al., 2022) were excluded because BBB scores were not assessed at 28 days and one study (Nie and Jiang, 2021) was excluded because the SCI model was transection model. Therefore, eight included studies (Yu et al., 2019; Huang et al., 2020; Chen et al., 2021; Huang L. et al., 2021; Huang W. et al., 2021; Zhang M. et al., 2021; He et al., 2022; Wang et al., 2022) were used for data analysis. The results of this network meta-analysis suggest that more studies evaluated EVs and miRNA-loaded EVs in the contusion model as compared with the compression model (Figure 9A). The forest plot (Figure 9B) and league table (Table 7) showed that EVs in contusion models (MD = 4.05; 95% CI: 2.23, 5.87; *p* < 0.01) and miRNA-loaded EVs in compression models (MD = 5.99; 95% CI: 1.16, 10.82; *p* < 0.01) and contusion models (MD = 6.83; 95% CI: 4.96, 8.63; *p* < 0.01) presented with a statistically significant advantage over PBS and that miRNA-loaded EVs in contusion models had a statistically significant advantage over the EVs in contusion models (MD = 2.78; 95% CI: 0.92, 4.58; *p* < 0.01). The differences were not statistically significant among the other comparisons. The SUCRA ranking graph showed that the miRNA-loaded EVs in contusion models (89.79%) ranked highest, followed by the miRNA-loaded EVs in compression models (77.15%), EVs in contusion models (49.41%), EVs in compression models (29.55%), and PBS (4.1%) (Figure 9C).

**Table 7:** League table of BBB scores in different models at 28 days after administration.

EVsCompression	1.73 (−3.41, 6.88)	3.68 (−1.1, 8.48)	4.52 (−0.69, 9.6)	−2.31 (−7.14, 2.5)
−1.73 (−6.88, 3.41)	EVsContusion	1.95 (−3.21, 7.06)	2.78 (0.92, 4.58)	−4.05 (−5.87, −2.23)
−3.68 (−8.48, 1.1)	−1.95 (−7.06, 3.21)	miREVsCompression	0.83 (−4.35, 5.95)	−5.99 (−10.82, −1.16)
−4.52 (−9.6, 0.69)	−2.78 (−4.58, −0.92)	−0.83 (−5.95, 4.35)	miREVsContusion	−6.83 (−8.63, −4.96)
2.31 (−2.5, 7.14)	4.05 (2.23, 5.87)	5.99 (1.16, 10.82)	6.83 (4.96, 8.63)	PBS

The gray color shades represent the different interventions and the bold values represent statistical significance.

### Inconsistency assessment

The following factors were not evaluated because the severity of illness at baseline, sex, weight, EVs' origins, and routes of administration were almost entirely consistent. Because there was no inconsistency in the multi-arm studies (Higgins et al., 2012) (all studies included in the network meta-analysis were multi-arm studies), the inconsistency could not be evaluated. The DIC difference between the consistency and inconsistency models was less than five, which showed that the results of the consistency model were robust (Supplementary File 3).

## Discussion

In summary, this systematic review and network meta-analysis of EVs derived from miRNA-modified MSCs for the treatment of SCI included data from 13 preclinical trials among a total of 396 rats.

### Summary of the evidence

#### Summary of comparison of EVs and miRNA-loaded EVs at different dosages

This study indirectly compared the efficacy of different doses of EVs and miRNA-loaded EVs in the treatment of SCI. We likewise indirectly compared the efficacy of EVs and miRNA-loaded EVs in different SCI models. The results of this network meta-analysis showed that miRNA-loaded EVs with 100 and 200 μg dosages statistically significantly improved hind limb motor function among SCI rats at the early stage (3 days), as compared with EVs with 100 and 200 μg dosages. However, in the middle and late stages (14 and 28 days), there were no statistically significant differences between EVs with 200 μg dosages and miRNA-loaded EVs with 100 μg dosages. In the late stages (28 days), there were no statistically significant differences between EVs with 100 μg dosages and miRNA-loaded EVs with 200 μg dosages. This may be related to the half-life of EVs. Although EVs presented with the properties of immune privilege, they were also inevitably removed by macrophages. The circulation half-life of the EVs has been reported as only ~30 min following intravenous administration (Yang et al., 2020). Therefore, it is likely that the results of this network meta-analysis were not statistically significant in the late stage of treatment because the EVs had been depleted. Moreover, the SUCRA ranking graph suggested that miRNA-loaded EVs at 200 μg dosages may be most effective for treatment in early, middle, and late stages. A study by Dumbrava et al. conducted in 2021 demonstrated that low-dose EVs promote nerve regeneration following ischemia, in contrast to high-dose EVs (Dumbrava et al., 2022). This phenomenon may be associated with the biodistribution of exogenous EVs *in vivo* following intravenous administration. Studies report that EVs are normally distributed in organs of the mononuclear phagocyte system and accumulate most in the liver, followed by the spleen, gastrointestinal tract, and lungs. However, some studies show that their distribution and clearance are affected by the size of the particles. For example, small EVs are less susceptible to extravasate through the discontinuous endothelium in the spleen, which makes them enter into the red pulp smoothly (Wiklander et al., 2015). Appropriate administered doses are likely safe and efficacious with EV therapy, while high doses may produce toxicity and ineffective therapeutic effects. These results guide physicians in selecting the proper therapeutic dose for EVs and miRNA-loaded EVs. The main EV delivery methods to damaged tissue include intrathecal and tail vein injection. Most studies have used the tail vein injection method because of its simplicity and minimally invasive (Yi and Wang, 2021). Interestingly, a study showed that EVs could improve significant locomotor recovery by intranasal injection (Guo et al., 2019). Furthermore, some scholars designed MSC-derived EV fibrin glue to treat spinal cord injury (Mu et al., 2021). Although EV delivery methods are important for the treatment of SCI, their extraction methods also play a key role. However, there is no uniform standard for the EV extraction methods. The most commonly used is ultracentrifugation. Various other techniques include density gradients, filtration, and precipitation (Thery et al., 2018).

#### Summary of comparison of EVs and miRNA-loaded EVs in different models

Additionally, the results of this network meta-analysis showed that miRNA-loaded EVs statistically significantly improved hind limb motor function among SCI rats at early stages in compression and contusion models, as compared with EVs. Moreover, there are results show that miRNA-loaded EVs statistically significantly improved hind limb motor function among SCI rats in the contusion model at the middle and late stages as compared with EVs. EVs could be used to treat spinal cord contusion, compression, and transection (Mu et al., 2021). Furthermore, they could treat spinal cord ischemia/reperfusion injury because of their ability to pass the blood-spinal barrier (Wu et al., 2022). A meta-analysis showed that EVs were more effective in treating contusion models as compared with compression models (Yi and Wang, 2021). Zhang found that MSC-derived EVs could inhibit inflammation and apoptosis by suppressing the PTEN and NF-κB signal (Zhang M. et al., 2021). Li found that MSC-derived EVs could activate ERK1/2, CREB, and STAT3, which play a critical part in the survival of neurons and the regeneration of axons (Li et al., 2018). Our network meta-analysis findings show that EVs derived from miRNA-modified MSCs can significantly improve hind limb motor function in SCI rats. Our study likewise found that miRNA-loaded EVs were more effective in treating contusion models than compression models. These results may be associated with the miRNA species found in EVs. Even so, these results could be partly explained by the fact that only three articles used the compression model and eight articles used the contusion model. On the other hand, it may be because the compression model is more unstable and prone to various biases, while the contusion model, as a classical SCI model, is more stable, so that the risk of various biases is relatively low in the process of modeling or treatment. However, direct comparative evidence is still needed to verify these results. Moreover, we found that miRNA-loaded EVs were more effective than EVs in treating SCI, consistent with the results of all included studies (Li et al., 2018, 2020; Yu et al., 2019; Huang et al., 2020; Chen et al., 2021; Zhang M. et al., 2021). The SUCRA ranking graph suggested that miRNA-loaded EVs may be most effective for the treatment of contusion models at the early, middle, and late stages. Therefore, miRNA-loaded EVs have tremendous therapeutic potential within SCI. Although there is no clear consensus with respect to SCI models, our study found that miRNA-loaded EVs are effective in compression and contusion models. These results meaningfully inform preclinical studies.

### Strengths and limitations

In terms of motor function scores on day 28 after SCI, this study found that miRNA-modified EVs therapy was effective in improving motor function scores compared with EVs alone or control therapy, and this is consistent with a recent study by Hu et al. (2021). However, our network meta-analysis also investigated motor function scores on day 3 and day 14 after SCI, whereas the recent meta-analysis only examined motor function scores on day 28. The results of our network meta-analysis indicate that miRNA-modified EVs therapy is more effective in improving motor function scores than EVs alone or control therapy in the early, middle and late stages of SCI. Therefore, the conclusions of our study are more comprehensive. To the best of our knowledge, this network meta-analysis is the first to compare the efficacy of different administered doses of EVs and miRNA-loaded EVs in different SCI models. Three nodes were selected for the network meta-analysis according to the pathological stage of the SCI (day 3 in the subacute stage, day 14 in the subacute and intermediate stages, and day 28 in the intermediate stage) (Rowland et al., 2008). Thus, we comprehensively compared the efficacy of administered EVs and miRNA-loaded EVs with respect to administered doses and different SCI models. The internationally recognized SYRCLE risk of bias tool was implemented to assay the quality of the included studies (Hooijmans et al., 2014). All included studies were of high methodological quality and had a low associated risk with respect to many items.

However, we acknowledge some limitations of this network meta-analysis. First, the anesthetic used for surgery and miRNA were not specified. Second, the number of included studies and their associated sample sizes are modest, which may pose a risk of bias. Third, all included studies had an unclear risk of blinding with respect to performance bias. Fourth, the BBB score was the only index of efficacy evaluation. This score is not sufficiently thorough or comprehensive, though it has remarkable objectivity. Fifth, using the SUCRA ranking graph to estimate ranking probabilities has inherent limitations and the results should be interpreted with caution (Salanti et al., 2011). Sixth, we only included SCI model of rats in the study. We had ever tried to extend the proposed analyses to the other studies, and to make a comparison with the results obtained in rats. However, we found that in addition to the SCI rats model, the other is SCI model in mice. The measure of motor function in mice is the Basso Mouse Scale for Locomotion (BMS), which is different from the Basso, Beattie, Bresnahan Locomotor Rating Scale (BBB) in rats. A study (Basso et al., 2006) showed that the recovery of motor function in mice is different from that in rats, and different behavioral indicators should be used. Moreover, the number of studies using the SCI mouse model was too small to conduct a network meta-analysis. Therefore, we included SCI model of rats in the study. Seventh, different miRNAs play different roles in SCI, including inhibiting inflammation, promoting axonal regeneration and promoting angiogenesis. However, in the complex environment *in vivo*, different miRNAs may exert direct or indirect effects on inhibiting inflammation, promoting axonal regeneration and promoting angiogenesis, which is not clear in all the studies included so far. Nevertheless, what we can identify is the macroscopic role they play (i.e., BBB motor function assessment), and therefore, we collected BBB functional score data to perform network meta-analysis. Even if these factors can affect the results of the network meta-analysis, this is inevitable. In conclusion, we consider that these factors may affect the results of the network meta-analysis, but the impact is not significant.

### Impact on future studies

Preclinical studies are essential for the application of interventions to clinical studies. Translating these studies in animal models to the delivery and effects of such an allogeneic source in human SCI therapies should focus on the difference between animal models and human SCI, such as the characteristics of disease, drug administration time, and drug efficacy. While it has shown that MSC-derived EVs could limit rejection in allogeneic transplantation, it is still necessary to carefully consider whether it is autologous or allogeneic when selecting their source (Lee et al., 2014). Many studies have compared the efficacy of EVs and miRNA-loaded EVs for the treatment of SCI. However, to the best of our knowledge, no trials have directly compared different EVs' doses of EVs and miRNA-loaded EVs. There are likewise no trials directly comparing the efficacy of EVs and miRNAs-loaded EVs in different SCI models. These respective efficacies can be compared in future studies. Moreover, the studies included in this network meta-analysis only measured efficacy indicators. None of the studies reported on safety indicators. Therefore, safety should be considered in future relevant preclinical studies in order to avoid adverse reactions upon clinical application.

## Conclusion

Our results indicate that miRNA-loaded EVs at 200 μg dosages may be the potential choice for the treatment of SCI. Moreover, miRNAs-loaded EVs may be the potential choice in contusion. However, there are some risks of bias in our included study. The mechanisms underlying the efficacy of EVs and the requirement for further clinical trials remain unclear. Therefore, additional preclinical trials are necessary to directly compare the efficacy of different therapeutic EVs' doses as well as the implementation of miRNA-loaded EVs in different SCI models. Such preclinical trials are beneficial in clinical transformation.

## Data availability statement

The original contributions presented in the study are included in the article/Supplementary material, further inquiries can be directed to the corresponding authors.

## Author contributions

ZY first presented the idea and designed the outline of the article. JR and ZL were responsible for all data extraction and analysis. The first version was written by ZY. FL was involved in article revision. The final version was revised by CC. All of authors reviewed and approved the final article proof for submission.

## Conflict of interest

The authors declare that the research was conducted in the absence of any commercial or financial relationships that could be construed as a potential conflict of interest.

## Publisher's note

All claims expressed in this article are solely those of the authors and do not necessarily represent those of their affiliated organizations, or those of the publisher, the editors and the reviewers. Any product that may be evaluated in this article, or claim that may be made by its manufacturer, is not guaranteed or endorsed by the publisher.
