# Supplementary material for: Efficacy of miRNA-modified mesenchymal stem cell extracellular vesicles in spinal cord injury: A systematic review of the literature and network meta-analysis
Source: Front Neurosci. 2022 Oct 5;16:989295. doi: 10.3389/fnins.2022.989295 (PMC9581233; doi:10.3389/fnins.2022.989295)
Supplement: Supplementary file 1 [file Table_1.DOCX]

**Additional file 1: Search query for databases**

**PubMed**

#1 "spinal cord injur*"[Title/Abstract] OR "Hemisection"[Title/Abstract] OR "contusion injury"[Title/Abstract] OR "dorsal column injury"[Title/Abstract] OR "complete transection"[Title/Abstract] OR "corticospinal tract injury"[Title/Abstract] OR "Paraplegia"[Title/Abstract] OR "Quadriplegia"[Title/Abstract] OR "Hemiplegia"[Title/Abstract] OR "tetraplegia"[Title/Abstract] OR "Monoplegia"[Title/Abstract] OR "spinal cord trauma"[Title/Abstract] OR "spinal cord transection"[Title/Abstract] OR "spinal cord laceration"[Title/Abstract] OR "spinal cord compromise"[Title/Abstract] OR "spinal cord lesion"[Title/Abstract] OR "spinal cord rupture"[Title/Abstract] OR "spinal cord contusion"[Title/Abstract] OR "spinal cord compression"[Title/Abstract] OR "spinal cord hemisection"[Title/Abstract] OR "traumatic myelopath*"[Title/Abstract] OR "spinal cord injur*"[MeSH Terms]

#2 "shedding vesicles"[Title/Abstract] OR "apoptotic bodies"[Title/Abstract] OR "exosome*"[Title/Abstract] OR "extracellular vesicles"[Title/Abstract] OR "nano sized vesicles"[Title/Abstract] OR "microvesicles"[Title/Abstract] OR "exosome*"[MeSH Terms]

#3 #1 AND #2

**Embase**

#1 'spinal cord injur*'/exp OR 'spinal cord injur*' OR 'spinal cord injur*':ab,ti OR 'Hemisection':ab,ti OR 'contusion injury':ab,ti OR 'dorsal column injury':ab,ti OR 'complete transection':ab,ti OR 'corticospinal tract injury':ab,ti OR 'Paraplegia':ab,ti OR 'Quadriplegia':ab,ti OR 'Hemiplegia':ab,ti OR 'tetraplegia':ab,ti OR 'Monoplegia':ab,ti OR 'spinal cord trauma':ab,ti OR 'spinal cord transection':ab,ti OR 'spinal cord laceration':ab,ti OR 'spinal cord compromise':ab,ti OR 'spinal cord lesion':ab,ti OR 'spinal cord rupture':ab,ti OR 'spinal cord contusion':ab,ti OR 'spinal cord compression':ab,ti OR 'spinal cord hemisection':ab,ti OR 'traumatic myelopath*':ab,ti

#2 'exosome*'/exp OR 'extracellular vesicles'/exp OR 'nano sized vesicles'/exp OR 'microvesicles'/exp OR 'shedding vesicles'/exp OR 'apoptotic bodies'/exp OR 'exosome*':ab,ti OR 'extracellular vesicles':ab,ti OR 'nano sized vesicles':ab,ti OR 'microvesicles':ab,ti OR 'shedding vesicles':ab,ti OR 'apoptotic bodies':ab,ti

#3 #1 AND #2

**Scopus:**

#1 ( TITLE-ABS-KEY ( "spinal cord injur*" )  OR  TITLE-ABS-KEY ( "Hemisection" )  OR  TITLE-ABS-KEY ( "contusion injury" )  OR  TITLE-ABS-KEY ( "dorsal column injury" )  OR  TITLE-ABS-KEY ( "complete transection" )  OR  TITLE-ABS-KEY ( "corticospinal tract injury" )  OR  TITLE-ABS-KEY ( "Paraplegia" )  OR  TITLE-ABS-KEY ( "Quadriplegia" )  OR  TITLE-ABS-KEY ( "Hemiplegia" )  OR  TITLE-ABS-KEY ( "tetraplegia" )  OR  TITLE-ABS-KEY ( "Monoplegia" )  OR  TITLE-ABS-KEY ( "spinal cord trauma" )  OR  TITLE-ABS-KEY ( "spinal cord transection" )  OR  TITLE-ABS-KEY ( "spinal cord laceration" )  OR  TITLE-ABS-KEY ( "spinal cord compromise" )  OR  TITLE-ABS-KEY ( "spinal cord lesion" )  OR  TITLE-ABS-KEY ( "spinal cord rupture" )   OR  TITLE-ABS-KEY ( "spinal cord contusion" )  OR  TITLE-ABS-KEY ( "spinal cord compression" ) OR  TITLE-ABS-KEY ( "spinal cord hemisection" ) OR  TITLE-ABS-KEY ( "traumatic myelopath*" ))

#2 ( TITLE-ABS-KEY ( "exosome*" )  OR  TITLE-ABS-KEY ( "extracellular vesicles" )  OR  TITLE-ABS-KEY ( "nano sized vesicles" )  OR  TITLE-ABS-KEY ( "microvesicles" )  OR  TITLE-ABS-KEY ( "shedding vesicles" ) OR  TITLE-ABS-KEY ( "apoptotic bodies" ))

#3 #1 AND #2

**The Cochrane Library**

#1 ("spinal cord injur*" OR "Hemisection" OR "contusion injury" OR "dorsal column injury" OR "complete transection" OR "corticospinal tract injury" OR "Paraplegia" OR "Quadriplegia" OR "Hemiplegia" OR "tetraplegia" OR "Monoplegia" OR "spinal cord trauma" OR "spinal cord transection" OR "spinal cord laceration" OR "spinal cord compromise" OR "spinal cord lesion" OR "spinal cord rupture" OR "spinal cord contusion" OR "spinal cord compression" OR "spinal cord hemisection" OR "traumatic myelopath*"):ti,ab,kw

#2 MeSH descriptor: [spinal cord injur*] explode all trees

#3 #1 OR #2

#4 ("exosome*" OR "extracellular vesicles" OR "nano sized vesicles" OR "microvesicles" OR "shedding vesicles" OR "apoptotic bodies"):ti,ab,kw

#5 MeSH descriptor: [exosome*] explode all trees

#6 #4 OR #5

#7 #3 AND #6

**Web of Science**

#1 TS=("spinal cord injur*" OR "Hemisection" OR "contusion injury" OR "dorsal column injury" OR "complete transection" OR "corticospinal tract injury" OR "Paraplegia" OR "Quadriplegia" OR "Hemiplegia" OR "tetraplegia" OR "Monoplegia" OR "spinal cord trauma" OR "spinal cord transection" OR "spinal cord laceration" OR "spinal cord compromise" OR "spinal cord lesion" OR "spinal cord rupture" OR "spinal cord contusion" OR "spinal cord compression" OR "spinal cord hemisection" OR "traumatic myelopath*")

#2 TS=("exosome*" OR "extracellular vesicles" OR "nano sized vesicles" OR "microvesicles" OR "shedding vesicles" OR "apoptotic bodies")

#3 #1 AND #2
